# Supplementary material for: Splice-Junction-Based Mapping of Alternative Isoforms in the Human Proteome
Source: Cell Rep. Author manuscript; Available in PMC 2020 Jan 15. (PMC6961840; doi:10.1016/j.celrep.2019.11.026)
Supplement: 2 [file NIHMS1546469-supplement-2.zip › DF1/PXD009737/TestisTrypsin/TestisTrypsin_50_IFI27L1_VIGGFAGTALGAWLGSPPSMGK.pdf]

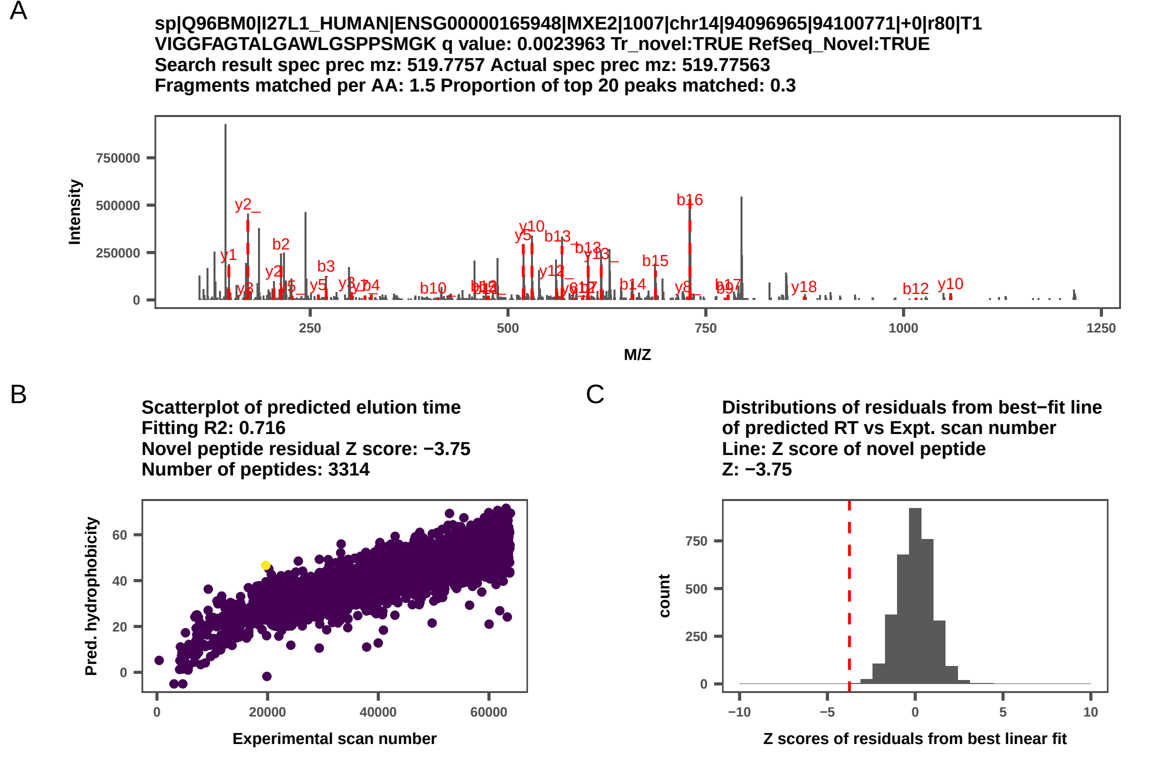
A
sp|Q96BM0|I27L1_HUMAN|ENSG00000165948|MXE2|1007|chr14|94096965|94100771|+0|r80|T1
VIGGFAGTALGAWLGSPPSMGK q value: 0.0023963 Tr_novel:TRUE RefSeq_Novel:TRUE
Search result spec prec mz: 519.7757 Actual spec prec mz: 519.77563
Fragments matched per AA: 1.5 Proportion of top 20 peaks matched: 0.3
Intensity
750000
500000
250000
0
250
500
750
1000
1250
M/Z
y1
y2_
b2
y2
b3
y5
b16
y10
b13
b15
b14
y18
b12
y10
b10
B
Scatterplot of predicted elution time
Fitting R2: 0.716
Novel peptide residual Z score: −3.75
Number of peptides: 3314
Pred. hydrophobicity
60
40
20
0
20000
40000
60000
Experimental scan number
C
Distributions of residuals from best−fit line
of predicted RT vs Expt. scan number
Line: Z score of novel peptide
Z: −3.75
count
750
500
250
0
−10
−5
0
5
10
Z scores of residuals from best linear fit
